# Supplementary figures and images for: Amyloid Properties of the FXR1 Protein Are Conserved in Evolution of Vertebrates
Source: Int J Mol Sci. 2022 Jul 20;23(14):7997. doi: 10.3390/ijms23147997 (PMC9319111; doi:10.3390/ijms23147997)

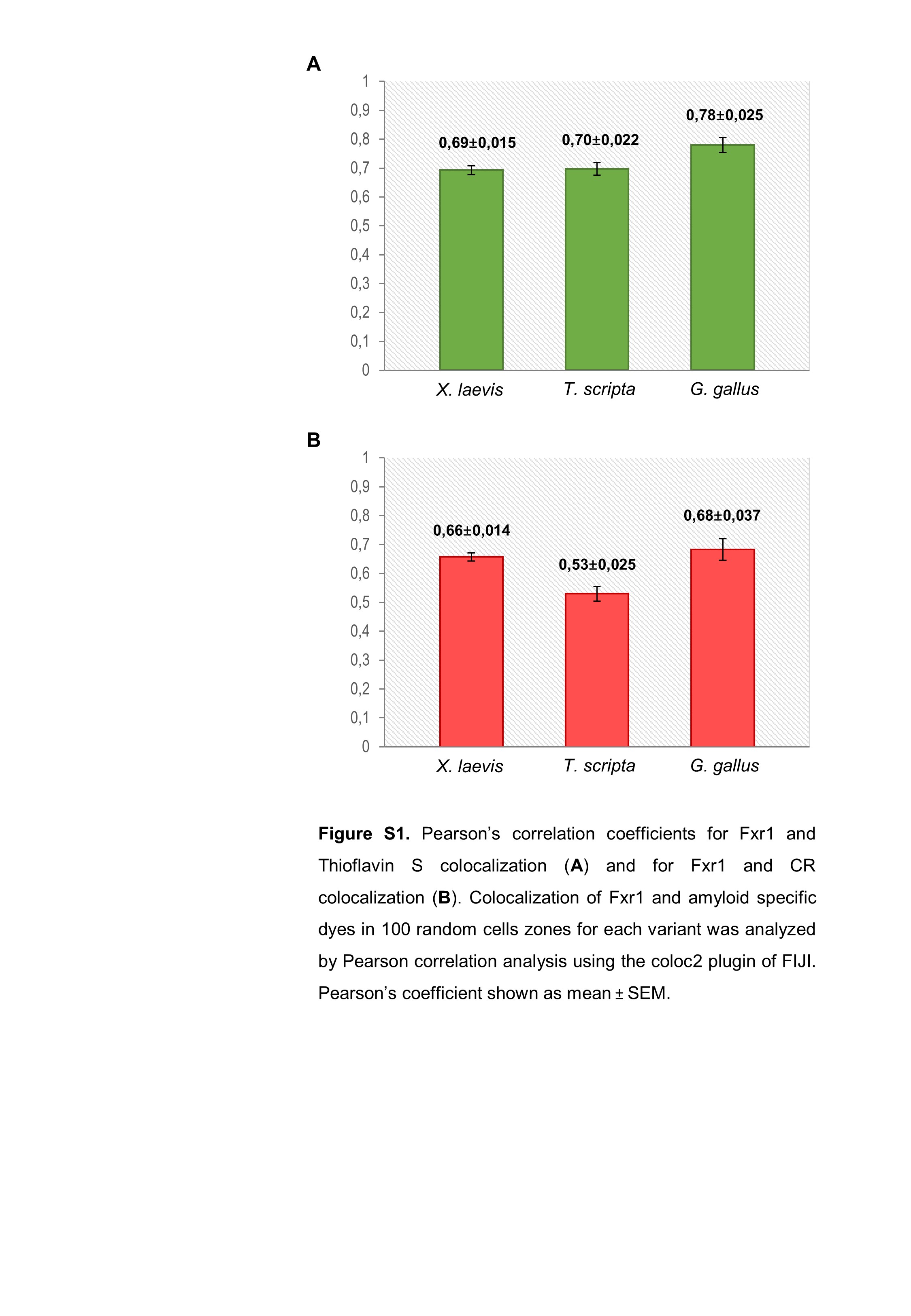

Supplement: Supplementary file 1 [file ijms-23-07997-s001.zip › Figure S1.jpg]

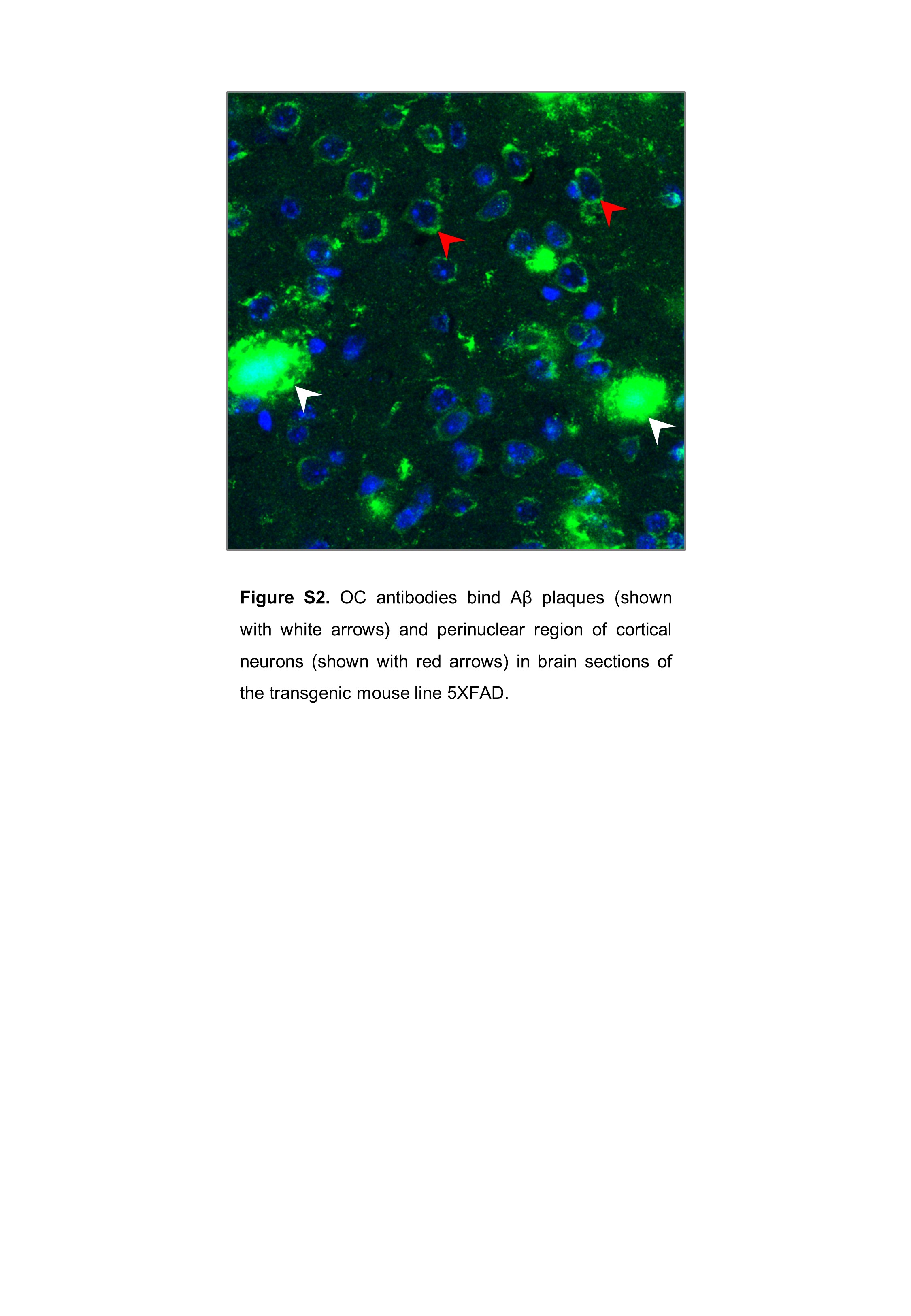

Supplement: Supplementary file 1 [file ijms-23-07997-s001.zip › Figure S2.jpg]

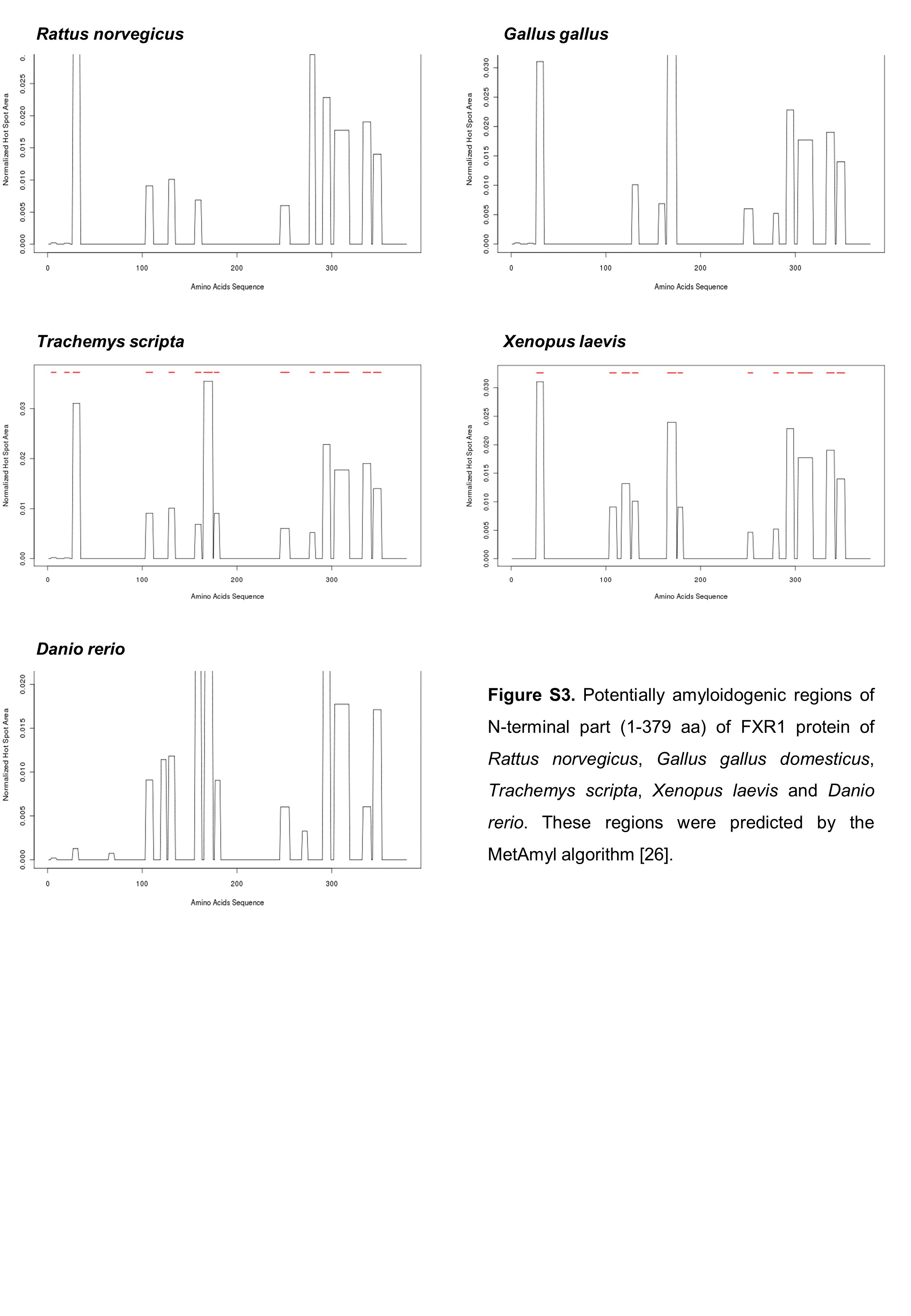

Supplement: Supplementary file 1 [file ijms-23-07997-s001.zip › Figure S3.jpg]
